# Salvage carbon dioxide transoral laser microsurgery for laryngeal cancer after (chemo)radiotherapy: a European Laryngological Society consensus statement

**DOI:** 10.1007/s00405-021-06957-5

**Published:** 2021-07-05

**Authors:** Cesare Piazza, Alberto Paderno, Elisabeth V. Sjogren, Patrick J. Bradley, Hans E. Eckel, Antti Mäkitie, Nayla Matar, Vinidh Paleri, Giorgio Peretti, Roberto Puxeddu, Miquel Quer, Marc Remacle, Vincent Vander Poorten, Isabel Vilaseca, Ricard Simo

**Affiliations:** 1 Department of Otorhinolaryngology—Head and Neck Surgery, ASST—Spedali Civili di Brescia, University of Brescia, Piazza Spedali Civili 1, 25123 Brescia, Italy; 2 Department of Medical, Surgical and Radiological Sciences and Public Health, University of Brescia, Brescia, Italy; 3 Department of Otolaryngology—Head and Neck Surgery, Leiden University Medical Centre, Leiden, The Netherlands; 4 Department of ORL-HNS, Queens Medical Centre Campus, Nottingham University Hospitals, Derby Road, Nottingham, UK; 5 Department of Oto-Rhino-Laryngology—Head and Neck Surgery, Klagenfurt General Hospital, Klagenfurt, Austria; 6 Department of Otorhinolaryngology—Head and Neck Surgery, University of Helsinki and HUS Helsinki University Hospital, Helsinki, Finland; 7 Department of Otolaryngology—Head and Neck Surgery, Saint Joseph University, Hotel Dieu de France Hospital, Beirut, Lebanon; 8 Head and Neck Unit, The Royal Marsden NHS Foundation Trust, London, UK; 9 Department of Otorhinolaryngology—Head and Neck Surgery, IRCCS Ospedale Policlinico San Martino, University of Genoa, Genoa, Italy; 10 Unit of Otorhinolaryngology—Head and Neck Surgery, Azenda Ospedaliero Universitaria, Presidio Ospedaliero Duilio Casula, Cagliari, Italy; 11 Department of Otolaryngology—Head and Neck Surgery, University Hospital de la Santa Creu i Sant Pau, Universitat Autonòma de Barcelona, Barcelona, Spain; 12 Department of Otolaryngology, Centre Hospitalier du Luxembourg, Luxembourg, Luxembourg; 13 Department of Otorhinolaryngology—Head and Neck Surgery, Leuven Cancer Institute, University Hospitals Leuven, Leuven, Belgium; 14 Department of Oncology, Section Head and Neck Oncology, KU Leuven, Leuven, Belgium; 15 Otorhinolaryngology Service, Hospital Clinic de Barcelona, Universitat de Barcelona, Barcelona, Spain; 16 Department of Otorhinolaryngology—Head and Neck Surgery, Head, Neck and Thyroid Cancer Unit, Guy’s and St Thomas Hospital NHS Foundation Trust, London, UK

**Keywords:** Carbon dioxide laser, Transoral laser microsurgery, Laryngeal cancer, Radiotherapy, Chemoradiation, Salvage surgery

## Abstract

**Purpose:**

To provide expert opinion and consensus on salvage carbon dioxide transoral laser microsurgery (CO_2_ TOLMS) for recurrent laryngeal squamous cell carcinoma (LSCC) after (chemo)radiotherapy [(C)RT].

**Methods:**

Expert members of the European Laryngological Society (ELS) Cancer and Dysplasia Committee were selected to create a dedicated panel on salvage CO_2_ TOLMS for LSCC. A series of statements regarding the critical aspects of decision-making were drafted, circulated, and modified or excluded in accordance with the Delphi process.

**Results:**

The expert panel reached full consensus on 19 statements through a total of three sequential evaluation rounds. These statements were focused on different aspects of salvage CO_2_ TOLMS, with particular attention on preoperative diagnostic work-up, treatment indications, postoperative management, complications, functional outcomes, and follow-up.

**Conclusion:**

Management of recurrent LSCC after (C)RT is challenging and is based on the need to find a balance between oncologic and functional outcomes. Salvage CO_2_ TOLMS is a minimally invasive approach that can be applied to selected patients with strict and careful indications. Herein, a series of statements based on an ELS expert consensus aimed at guiding the main aspects of CO_2_ TOLMS for LSCC in the salvage setting is presented.

## Introduction

Radiotherapy (RT) and chemoradiation (CRT) are well-established treatments for laryngeal squamous cell carcinoma (LSCC), with oncologic outcomes comparable with those obtained by surgery when appropriate patient selection has been accomplished. However, local recurrence after primary RT is not a rare event even in early–intermediate LSCC, ranging from 5 to 20 and from 25 to 30% in cT1 and cT2 lesions, respectively [1–5]. This poses a significant issue in patient management given the limited salvage treatment options after (C)RT. Laryngeal re-irradiation is rarely possible due to poor oncological and functional results [6–8], and hence the need to opt for other treatments. Open-neck conservative options are hampered by RT-induced tissue modifications that frequently lead to complications and unpredictable recurrence patterns, possibly reducing the rates of laryngeal preservation and disease control. This prevented their widespread acceptance and application, while different groups confirmed their effectiveness in very selected cases even in the rescue setting [9–11]. As a consequence, in most instances, total laryngectomy is still considered the standard salvage treatment for patients previously treated with (C)RT experiencing LSCC recurrence. However, in such cases, ensuing complications and unavoidable functional sequelae (i.e., loss of physiologic phonation and swallowing impairment) almost invariably lead to a decreased quality of life.

For these reasons, carbon dioxide transoral laser microsurgery (CO_2_ TOLMS) definitively represents, when feasible, an extremely attractive option [12]. Several authors have confirmed the feasibility and oncological soundness of salvage CO_2_ TOLMS (Table 1) [13–28]. However, the suggested indications are highly variable and shared guidelines strongly needed. Therefore, the European Laryngological Society (ELS) selected an expert panel to discuss and reach consensus on key statements regarding preoperative management, treatment indications, and follow-up of LSCC after (C)RT. These statements represent the ensuing opinion coming from such a consensus and, in our view, could potentially guide the decision-making process in the salvage CO_2_ TOLMS scenario.

**Table 1:** Oncologic outcomes of CO_2_ TOLMS in the salvage setting reported in the recent literature and including only series with 5-year results

Authors	Year	No. of patients	T-categories	5-year DSS	5-year OS
Quer et al. [14]	2000	24	rT1-rT2	100%	76%
Steiner et al. [17]	2004	34	rT1-rT4	86%	53%
Ansarin et al. [19]	2007	37	rTis-rT2	–	86%
Roedel et al. [22]	2010	53	rT1-rT3	68.6%	53.3%
Han et al. [23]	2012	18	rT1-rT2	90%	84.3%
Del Bon et al. [24]	2012	35	rT1-rT2	94%	91%
Meulemans et al. [28]	2018	33	rT1-rT3	95.5%	80.3%

## Methods

Members of the panel were selected from the ELS Committee on Laryngeal Cancer and Dysplasia (9 members, one of which declined participation) and other ELS members with proven experience in CO_2_ TOLMS until reaching the number of 15. This number was agreed to ensure enough variation and, at the same time, allow an odds ratio for which a consensus was reached.

The initial statements were drafted by two authors (C.P. and A.P.) based on their personal experience and relevant literature. A modified Delphi survey was constructed for distribution throughout the group of authors (C.P., A.P., E.V.S., P.J.B, H.E.E., A.M., N.M., V.P., G.P., R.P., M.Q., M.R., V.V.P., I.V., and R.S.) to determine the consensus for each provisory recommendation [29].

### Modified Delphi process

The modified Delphi process was utilized to determine which statements achieved full consensus. Through this method, each expert on the panel expressed his/her opinion for each proposed statement using a dedicated online survey. Responses for the modified Delphi survey were collected electronically. Results were analyzed using a Likert scale ranging from 1 to 9, utilizing the following anchor points: 1 (strongly disagree), 3 (disagree), 5 (neutral), 7 (agree), and 9 (strongly agree). Statements were defined as achieving full consensus if there was a mean score of 7 or greater, and no outlier responses (scores lower than 5). Statements were considered achieving borderline consensus (thus needing modification and supplementary evaluation) when scores ranged between 6 and 7, or more than 7 with outlier responders. Statements were considered without consensus (and therefore deleted) when the score was less than 6, regardless of the presence or not of outliers.

The process required a total of three rounds. In the first round, responses were double-blinded (to both responders and analyzers). In this phase, the statements without consensus were deleted, while those with borderline consensus were selected, modified to better specify the rationale of the items, and re-evaluated in a second round. The second round was single-blinded (only to responders) to allow subsequent feedback from those in disagreement (scores lower than 5). The third round consisted in personal communication among panel members to negotiate a final consensus.

## Results

Response rates to the survey were 100% (15 of 15 panelists) for the first and second rounds, while only two members of the panel were involved in the third round.

### First round

A total of 20 statements were drafted and submitted to all members. Among those, 13 reached a full consensus in this phase (mean score, 8.2; range 7.6–8.7). Conversely, one statement did not reach consensus (score, 5.9) and was therefore deleted, while 6 reached a borderline consensus due to insufficient score in 3 (mean score, 6.4; range 6.1–6.7), or outlier responses in 3. Statements reaching borderline consensus were subsequently modified and re-submitted in the second round.

### Second round

A total of 6 statements, modified from the first round, were submitted to all members. Five reached full consensus (mean score, 7.9; range, 7.6–8.4), while one reached borderline consensus due to outlier responses by 2 panelists. The statement not reaching full consensus was further discussed in the third round.

### Third round

Consensus on the last statement was reached by direct discussion with the two experts in disagreement. After accurate evaluation and contextualization of the statement, consensus was reached without the need to modify the statement itself.

## Discussion and recommendations

### Diagnostic work-up

#### Assessment of pre-(C)RT staging

- The pre-(C)RT staging of the disease needs to be carefully assessed to determine the best salvage treatment. Recurrent lesions after RT originating from a primary tumor initially categorized as cT1 or cT2 without impairment of vocal fold mobility and/or anterior transcommissural extension can be considered for salvage CO_2_ TOLMS.
- Tumors initially categorized as cT2 with impaired vocal cord mobility, anterior transcommissural extension, cT3 for vocal cord/arytenoid fixation, or thyroid cartilage erosion, and failed after (C)RT should be considered suboptimal candidates for salvage CO_2_ TOLMS.

These considerations are related to the fact that tumor response to (C)RT does not induce a homogeneous concentric shrinkage of the lesion; instead, multiple resistant tumor foci may residue in otherwise healthy/cicatricial tissues [30]. The possibility of underestimation of the T category prior to (C)RT should be also taken into account. Consequently, safe resection margins can only be obtained by planning the excision in consideration of the initial tumor extent before (C)RT. This cannot be obtained if the primary tumor was not safely resectable by CO_2_ TOLMS in the first instance, as is especially the case for cT3 lesions with vocal cord/arytenoid fixation or thyroid cartilage erosion [31].

### Clinical and office-based assessment of rT

- Preoperative evaluation should be performed by high-definition videolaryngoscopy, possibly using biologic endoscopy techniques such as Narrow Band Imaging (NBI), Storz Professional Image Enhancement System (SPIES), I-Scan, or others.
- Preoperative prediction of laryngeal exposure can be of help in treatment planning to identify patients at a high risk of difficult laryngeal exposure.

Preoperative staging is a crucial step in planning salvage CO_2_ TOLMS. Underestimation of rT-category is a frequent issue due to diagnostic difficulties in the post-(C)RT setting [22]. In particular, RT-related inflammation and mucosal alterations may be a significant confounding factor leading to late identification of recurrent mucosal lesions. Endoscopy plays a pivotal role in diagnostic assessment of recurrence. Post-RT tissue modifications should be taken into account and distinguished from tumor relapse. In line with ELS recommendations for the follow-up of patients treated for LSCC [32], high-definition videolaryngoscopy, possibly integrated with video recording, storage of images, and use of bioendoscopy, is the most accurate clinical evaluation tool. This is especially true after (C)RT, thanks to the high accuracy of bioendoscopy in differentiating between neoplastic disease and post-RT inflammatory/cicatricial changes, even though mainly limited to the superficial aspect of the mucosal lining and needing a longer learning curve than that observed for naïve LSCC [33, 34]. Evaluation of vocal fold mobility is also essential to direct treatment indications. Impaired vocal fold mobility or arytenoid fixation can result from RT-induced damage of the vocal muscle or crico-arytenoid joint. However, it can also be a sign of deep tumor infiltration that needs to be discerned by specific radiologic evaluation [35].

Good laryngeal exposure is an essential consideration in previously irradiated patients and can be compromised because of reduced neck flexibility and tissue elasticity. The Laryngoscore and the mini-Laryngoscore are preoperative tools aimed at predicting laryngeal exposure by analyzing patient characteristics [36–39]. The Laryngoscore includes previous (C)RT as a negative factor for intraoperative exposure, and its influence should be considered together with other anatomical variables.

### Radiologic evaluation

- Cross-sectional imaging is warranted in all patients with recurrent LSCC after (C)RT. Magnetic resonance (MR) should be preferred in view of its capability to better distinguish between inflammation, scar, and neoplastic tissue.
- Preoperative imaging by chest computed tomography (CT) scan, total body CT scan, or positron emission tomography (PET)/CT scan should be employed to exclude distant metastasis and synchronous tumors.

Submucosal tumor spread of recurrent LSCC is a frequent occurrence, thus hindering detection through endoscopic techniques. For these reasons, state-of-the-art radiologic imaging should be employed. MR offers higher contrast resolution than CT, and is particularly helpful in distinguishing tumor infiltration from peritumoral inflammation and depicting cartilage invasion [40]. This is especially true using surface coils directly applied to the neck, designed to provide improved signal-to-noise ratios by limiting the spatial extent of the excitation and reception [35].

Finally, in the setting of persistent or recurrent LSCC after (C)RT, systemic imaging should be employed to rule out distant metastasis. PET/CT scan, carried out at least 12 weeks after treatment, demonstrated an adequate diagnostic potential in LSCC and head and neck cancer in general [41–43]. However, its effectiveness in detecting occult nodal metastases in recurrent LSCC remains debated [44]. Alternative imaging protocols (i.e., total body CT or chest CT scan) may be considered, especially in low-risk patients [45], in view of their minor cost and broader availability.

### Evaluation under general anesthesia

- Tumor evaluation with high-definition rigid endoscopy (under white light and NBI/SPIES/I-Scan) and biopsy under general anesthesia should be considered in all patients with an indication to salvage CO_2_ TOLMS and indeterminate findings at the office-based evaluation.
- Evaluation of the real exposure and transoral full visibility of the entire tumor through the laryngoscope should ideally precede CO_2_ TOLMS of any rT to improve treatment planning and patient counseling.

Considering the potential difficulties and confounding factors related to office-based endoscopy after (C)RT, panendoscopy under general anesthesia should be performed with a very low threshold in all cases with indeterminate or suspicious findings at endoscopic and/or radiologic evaluation. Random biopsies should be avoided because of their limited diagnostic potential and risk of complications (e.g., soft-tissue edema, and chondritis with possible chondronecrosis). Conversely, biopsies should be precisely targeted (eventually following laser incision to reach submucosal tissues) to suspicious areas found using biologic endoscopy techniques.

Salvage CO_2_ TOLMS requires optimal laryngeal exposure given the need for a more extensive resection and difficult discrimination of tumor boundaries. In patients with a high risk of difficult laryngeal exposure at preoperative evaluation, assessment of exposure under general anesthesia is essential to direct the subsequent treatment approach. In all cases, optimal laryngeal exposure with a large-bore laryngoscope should be obtained, allowing adequate visualization up to the anterior commissure. Excellent laryngeal exposure is an absolute prerequisite for salvage CO_2_ TOLMS (even more than in the primary setting), since safe resection margins can usually be obtained only by more enlarged procedures (Type III–VI cordectomy) and accurate microscopic assessment. Patients presenting suboptimal exposure should be evaluated for alternative approaches regardless of their rT-category.

### Treatment indications

#### Glottic recurrence

- Glottic LSCCs recurring after (C)RT and categorized as rcTis-T2 with normal vocal fold mobility can be considered for salvage CO_2_ TOLMS. However, pre-treatment staging and laryngeal exposure (see "Diagnostic work-up") should be carefully taken into account.
- Glottic rT1b with anterior commissure involvement and rT2 with anterior transcommissural extension should be considered for CO_2_ TOLMS only in the presence of optimal laryngeal exposure.

Various authors described the oncologic outcomes of salvage CO_2_ TOLMS and proposed different indications in relation to complications and survival results [13–28]. Most series included rTis-T2 tumors; however, in some cases, indications were extended up to rT4a lesions [17, 22]. Treatment of T3-T4a glottic LSCC has been described in both primary and salvage CO_2_ TOLMS, but requires careful selection and significant surgical and technical expertise, being associated with unpredictable functional outcomes, notwithstanding the frequent need for complementary (C)RT, which is clearly not repeatable in a salvage setting. Moreover, rT3-T4a lesions, as well as rT2 with impaired vocal fold mobility or anterior transcommissural extension, may have unexpected patterns of spread due to their deep infiltration, thus increasing the risk of local recurrence. In this setting, the results are strongly operator-dependent, and this factor prevents large-scale treatment adoption with homogeneous oncologic results. Furthermore, extensive removal of the perichondrium and resection of the cartilage can lead to impaired wound healing, chondronecrosis, and necrosis of soft tissues. These complications often result in severe functional impairment, impacting airway patency and swallowing, and may ultimately require total laryngectomy. The same holds true when considering recurrent LSCCs originating from advanced primary lesions (i.e., cT3-T4). Adequate treatment of these lesions including the pre-(C)RT tumor margins would require a resection extending to the perichondrium or cartilage, thus incurring in the abovementioned issues.

As in the primary setting, management of tumors with anterior commissure involvement remains a debated issue. In this regard, oncologic results in the salvage setting are variable and their evaluation is severely impaired by the small sample size of the cohorts reported [15, 17, 19, 22]. In fact, some authors confirm anterior commissure involvement as a risk factor for recurrence, while others understate its risk potential. However, in view of the complex anatomical and technical management of this subsite [46], optimal laryngeal exposure should always be ensured before embarking into a salvage CO_2_ TOLMS addressing the region. This recommendation has also been confirmed by Steiner and coworkers, highlighting a lower laryngeal preservation rate in patients with anterior commissure involvement and suboptimal laryngeal exposure [17].

#### Supraglottic recurrence

- Supraglottic LSCCs categorized as rcTis-T2 can be effectively treated by salvage CO2 TOLMS.

As for glottic LSCC, various authors have described the technical feasibility of transoral supraglottic resection up to rT3 lesions [13, 14, 21, 25, 26]. However, the fibro-cartilaginous laryngeal framework does not effectively stem bulky T3 and T4a tumors that invariably require resection of the hyoid bone, thyrohyoid membrane, thyroid cartilage, and soft tissues of the neck. Surgery in this setting becomes particularly challenging, hampering its widespread adoption with satisfying oncologic outcomes. The ensuing functional results are often suboptimal and worsened by frequent postoperative and long-term complications (e.g., chronic aspiration, pneumonia, chondronecrosis, and soft-tissue necrosis).

rcTis-T2 tumors may be more easily approached and treated by salvage CO_2_ TOLMS. However, patient selection plays a crucial role in treatment planning. Functional outcomes are invariably worse than in the primary setting, and patients should be able to tolerate chronic subclinical aspiration and a lengthy swallowing rehabilitation program. In particular, extensive resection of the aryepiglottic fold with arytenoidectomy should be avoided given the extremely high risk of postoperative acute and chronic aspiration [47].

#### Regional recurrence

- Concomitant local (rT > 1) and regional (rN > 1) recurrence after (C)RT should be considered a suboptimal indication to salvage CO_2_ TOLMS.

Salvage CO_2_ TOLMS should not be considered as a first-line treatment for patients with extensive loco-regional recurrence of LSCC because of different factors. First of all, the presence of regional recurrence should be regarded as a sign of potential underestimation of the true primary and/or recurrent tumor extension. Lateral neck metastasis may be related to undetected deep tumor extension and lympho-vascular invasion.

Moreover, it is not possible to adequately address the lymphatic vessels connecting the tumor with pathologic lymph nodes (the so-called “T-N tract”) through a purely transoral approach. In-transit metastatic cells may be left in the soft tissues between the endoscopic surgical field and the neck dissection specimen. Finally, patients treated by concomitant CO_2_ TOLMS and neck dissection have a significant risk of complications (such as fistula formation or subcutaneous emphysema) and postoperative laryngeal edema. In this view, the “cost–benefit ratio” of salvage CO_2_ TOLMS tends to increase dramatically in case of advanced lateral neck recurrence (rN > 1), both in terms of oncologic results and complication rate.

### Postoperative management and complications

- Prophylactic antibiotic therapy should be used to prevent postoperative infection/chondritis/chondronecrosis.
- Resumption of oral feeding is usually straightforward after glottic procedures; however, in supraglottic resections, there is often a need for nasogastric feeding tube for some days.
- Patients should be carefully monitored to detect early signs of perichondritis, chondritis, or chondronecrosis so that appropriate therapeutic measures can be instigated.

Salvage CO_2_ TOLMS can lead to reduced sensitivity and mobility of the larynx, resulting in an increased risk of aspiration during the early postoperative period. Patients undergoing supraglottic resections (particularly if reaching the aryepiglottic fold and/or the arytenoid) or extensive glottic procedures are at higher risk of aspiration and therefore prolonged use of a nasogastric feeding tube [47].

Perichondral damage following CO_2_ TOLMS may allow bacteria to gain access to the cartilage with possible infection. This may lead to perichondritis, an inflammatory process of the perichondrium that precedes chondritis and chondronecrosis. In hypovascularized and irradiated tissues, infection initiates a vicious cycle leading to tissue damage and hypoxia, compromised vascularity, and further progression of the infective/inflammatory process [48, 49]. Consequently, chondronecrosis is significantly more likely in patients who received high-dose RT combined with exposure or disruption of the perichondrium. For this reason, prophylactic antibiotic therapy may be helpful in patients receiving resections exposing the perichondrium, while no data are yet available on the utility of this aid.

Once developed, laryngeal chondronecrosis is often irreversible. Total laryngectomy is frequently required because of life-threatening bleeding, laryngeal framework collapse, and ensuing airway obstruction. Chondronecrosis should be monitored and evaluated according to the grading system proposed by Chandler et al. [50]. Grades I and II are common post-RT changes and typically respond favorably to conservative treatments (i.e., humidification, voice restraint, discontinuation of smoking, and antibiotics), while Grade III and IV reactions are more severe and have less favorable outcomes. Perichondritis, chondritis, and chondronecrosis may respond well to hyperbaric oxygen therapy, even though persistent tumor should be absolutely ruled out before considering this treatment to avoid hyperbaric oxygen-induced tumor acceleration [51, 52]. However, Grades III and IV are significantly less likely to be successfully treated by a conservative approach.

Complications of salvage CO_2_ TOLMS reported in the recent literature are summarized in Table 2.

**Table 2:** Complications reported in the recent literature

Authors	Year	No. of patients	Reported complications
Quer et al. [14]	2000	24	Laryngeal stenosis (*N* = 2)
De Gier et al. [15]	2001	44	Chondritis (*N* = 2)
Steiner et al. [17]	2004	34	Synechiae (*N* = 3), aspiration pneumonia (*N* = 1), chondronecrosis (*N* = 1), laryngeal stenosis (*N* = 1)
Puxeddu et al. [18]	2004	16	None
Ansarin et al. [19]	2007	37	Laryngeal stenosis (*N* = 4)
Roedel et al. [22]	2010	53	Laryngeal edema (*N* = 5), synechiae (*N* = 4), laryngeal stenosis (*N* = 3), postoperative bleeding (*N* = 2), chondronecrosis (*N* = 1)
Han et al. [23]	2012	18	Excessive granulation tissue (*N* = 3), temporary lingual numbness (*N* = 2), temporary hypoglossal palsy (*N* = 1)
Del Bon et al. [24]	2012	35	Chondronecrosis (*N* = 2), chondritis (*N* = 1), postoperative bleeding (*N* = 1)
Abouyared et al. [26]	2014	52	Prolonged postoperative pain (*N* = 13), chondronecrosis (*N* = 12)
Fink et al. [27]	2016	42	Laryngeal edema (*N* = 1)

### Functional outcomes

- Tracheotomy and gastrostomy tube placement are infrequently, and usually only temporarily, needed in the salvage CO_2_ TOLMS setting.
- Vocal outcomes after salvage CO_2_ TOLMS are usually inferior to those obtained in the primary setting. Moderate or severe dysphonia should be expected.
- A slower and more gradual resumption of swallowing should be expected in supraglottic resections compared with purely glottic procedures.

The length of feeding tube dependency and chronic aspiration rate are significantly higher in salvage CO_2_ TOLMS [53]. Nevertheless, according to a recent systematic review, postoperative tracheotomy and gastrostomy in patients treated by CO_2_ TOLMS were needed only in 2.3% and 6.6% of patients, respectively [54]. Furthermore, several studies have reported higher gastrostomy tube dependency rates in supraglottic recurrences compared to glottic ones [25, 26]. However, the number of patients involved was not sufficient for precise and reliable comparisons.

Objective data assessing vocal outcomes in salvage CO_2_ TOLMS are scarce and heterogeneous. In general, the results seem to be inferior to those in the primary setting, while a small study providing a direct comparison did not find significant differences [24]. In a study by Puxeddu and coworkers, postoperative perceptual voice evaluation (grade, roughness, breathiness, asthenia, and strain score) showed mild, moderate, and severe dysphonia in 12.5%, 25%, and 37.5% of patients, respectively. Furthermore, videolaryngostroboscopy showed incomplete glottic closure in 56% of patients [18].

### Follow-up

- Follow-up policy should strictly observe guidelines provided by the ELS, with particular attention to the indications after (C)RT.

Follow-up has a critical role in the overall management of patients treated by salvage CO_2_ TOLMS. In fact, recurrences are more frequent and less easily detected than in the primary setting. All measures described in the “Diagnostic work-up” section should be undertaken when evaluating a newly developed lesion or functional alteration. In adjunction, the development of treatment-related complications should also be monitored in the medium and long terms.

The ELS has proposed a series of recommendations for the follow-up of patients treated for LSCC to provide an up-to-date, evidence-based protocol that is meaningful and applicable to all European health care systems [32]. These recommendations represent an optimal framework from which to structure patient follow-up after salvage CO_2_ TOLMS.

## Conclusions

Management of recurrent LSCC after (C)RT is particularly challenging and requires careful evaluation to select the optimal therapeutic option according to the characteristics of the patient and tumor. In this setting, CO_2_ TOLMS has the crucial role to allow a conservative and minimally invasive approach even after failure of primary non-surgical therapy. However, when CO_2_ TOLMS is the preferred salvage treatment, patient selection is essential to achieve satisfying oncologic and functional results. Therefore, diagnostic work-up, treatment indications, and postoperative management should be optimized according to the available evidence. In consideration of the scarcity of data in the current literature, the statements collected in this manuscript and reflecting the expert opinion of a panel of European laryngologists may significantly help this process and favor standardization in management of patients treated by salvage CO_2_ TOLMS.
